# Supplementary material for: IL-2 delivery to CD8+ T cells during infection requires MRTF/SRF-dependent gene expression and cytoskeletal dynamics
Source: Nat Commun. 2024 Sep 11;15:7956. doi: 10.1038/s41467-024-52230-8 (PMC11391060; doi:10.1038/s41467-024-52230-8)
Supplement: Supplementary file 1 — Supplementary Information [file 41467_2024_52230_MOESM1_ESM.pdf]

SUPPLEMENTARY INFORMATION

IL-2 delivery to CD8$^+$ T cells during infection requires MRTF/SRF-dependent gene expression and cytoskeletal dynamics

SUPPLEMENTARY FIGURES AND LEGENDS

Figure S1. Phenotypic analysis of OT-I *Srf*$^{-/-}$ CD8$^+$ T cells

Figure S2. *Srf* is required for effective SLEC accumulation.

Figure S3. Phenotypic analysis of naïve OT-I *Mrtfab*$^{-/-}$ CD8$^+$ T cells

Figure S4. OT-I *Mrtfab*$^{-/-}$ cells localisation after LM-OVA infection

Figure S5. IL-2 signalling is defective in OT-I Srf$^{-/-}$ during infection

Figure S6. Responses to exogenous IL-2 in *Srf*$^{-/-}$ and *Mrtfab*$^{-/-}$ cells

Figure S7. Defective cluster formation by OT-I *Mrtfab*$^{-/-}$ cells .

Figure.S8. Gene expression deficits in OT-I *Mrtfab*$^{-/-}$ cells

Figure S9. MRTF-dependent F-actin assembly in homotypic clustering

Figure S10. Gating strategy

SUPPLEMENTARY DATASETS

Supplementary Data 1 **Gene expression in CD8$^+$ T cells**

Supplementary Data 2 **Gene cluster analysis**

SOURCE DATA

Source Data File 1 **Numerical source data for all Figures**

Source Data File 2 **Immunoblot source data for all Figures**

Figure S1

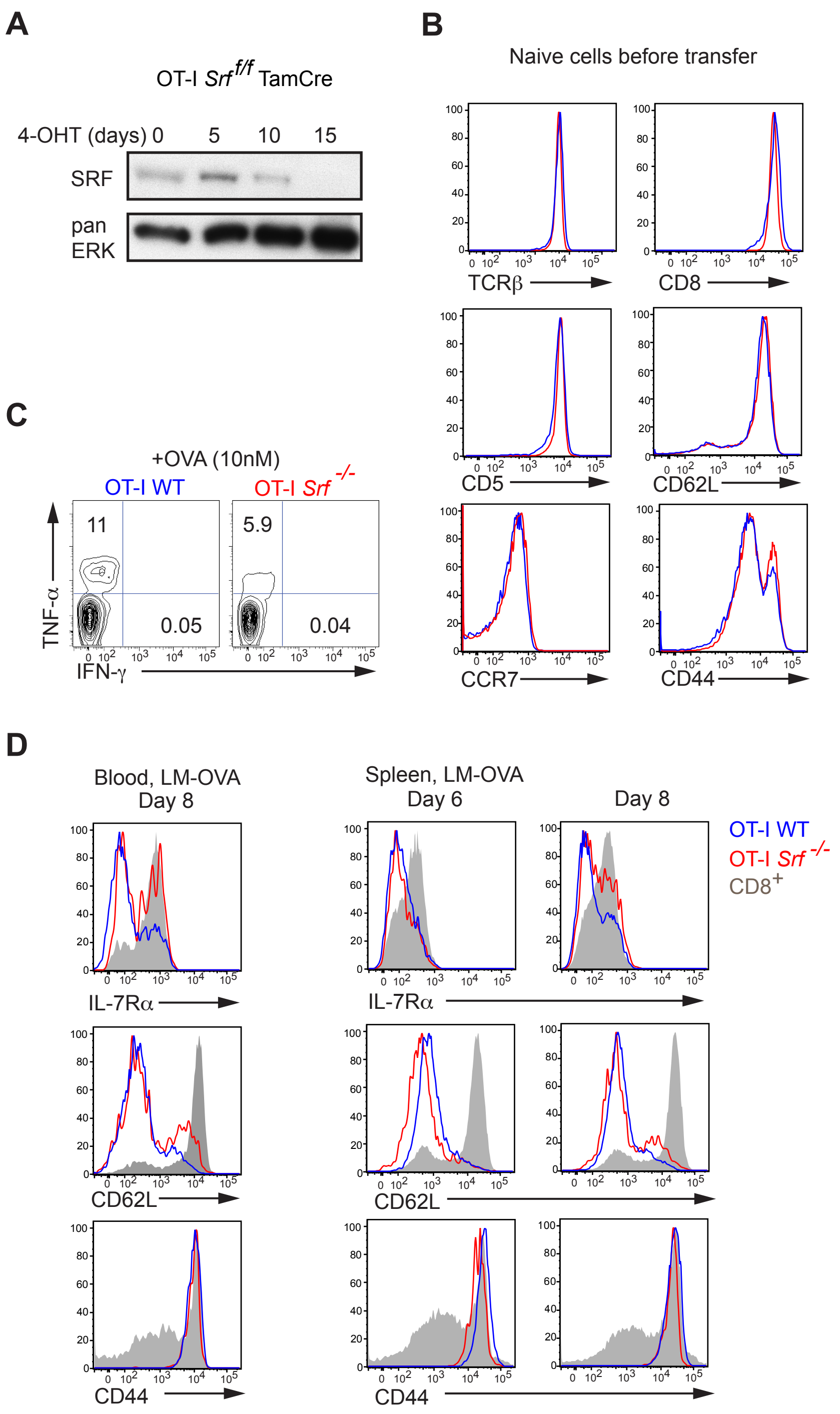

A
OT-I Srf f/f TamCre
4-OHT (days) 0 5 10 15
SRF
pan ERK
B
Naive cells before transfer
TCRβ
CD8
CD5
CD62L
CCR7
CD44
C
+OVA (10nM)
OT-I WT
OT-I Srf -/-
11
0.05
5.9
0.04
TNF-α
IFN-γ
D
Blood, LM-OVA
Day 8
Spleen, LM-OVA
Day 6
Day 8
OT-I WT
OT-I Srf -/-
CD8+
IL-7Rα
CD62L
CD44

Figure S1. Phenotypic analysis of OT-I *Srf*$^{-/-}$ CD8^{+} T cells

(A) Time course (days) of SRF expression in tamoxifen-fed animals.

(B) OT-I WT and OT-I *Srf*$^{-/-}$ CD8^{+} T cells were MACS-purified from the spleens of tamoxifen-treated bone marrow reconstituted mice and stained for TCRα, CD8, CD5, CCR7, CD62L and CD44 expression.

(C) Purified OT-I cells were stimulated for 5h with SIINFEKL OVA peptide and analysed for intracellular IFN-γ and TNF-α expression.

(D) IL-7Rα CD62L and CD44 expression *in vivo* after LM-OVA infection in spleen and blood.

Figure S2

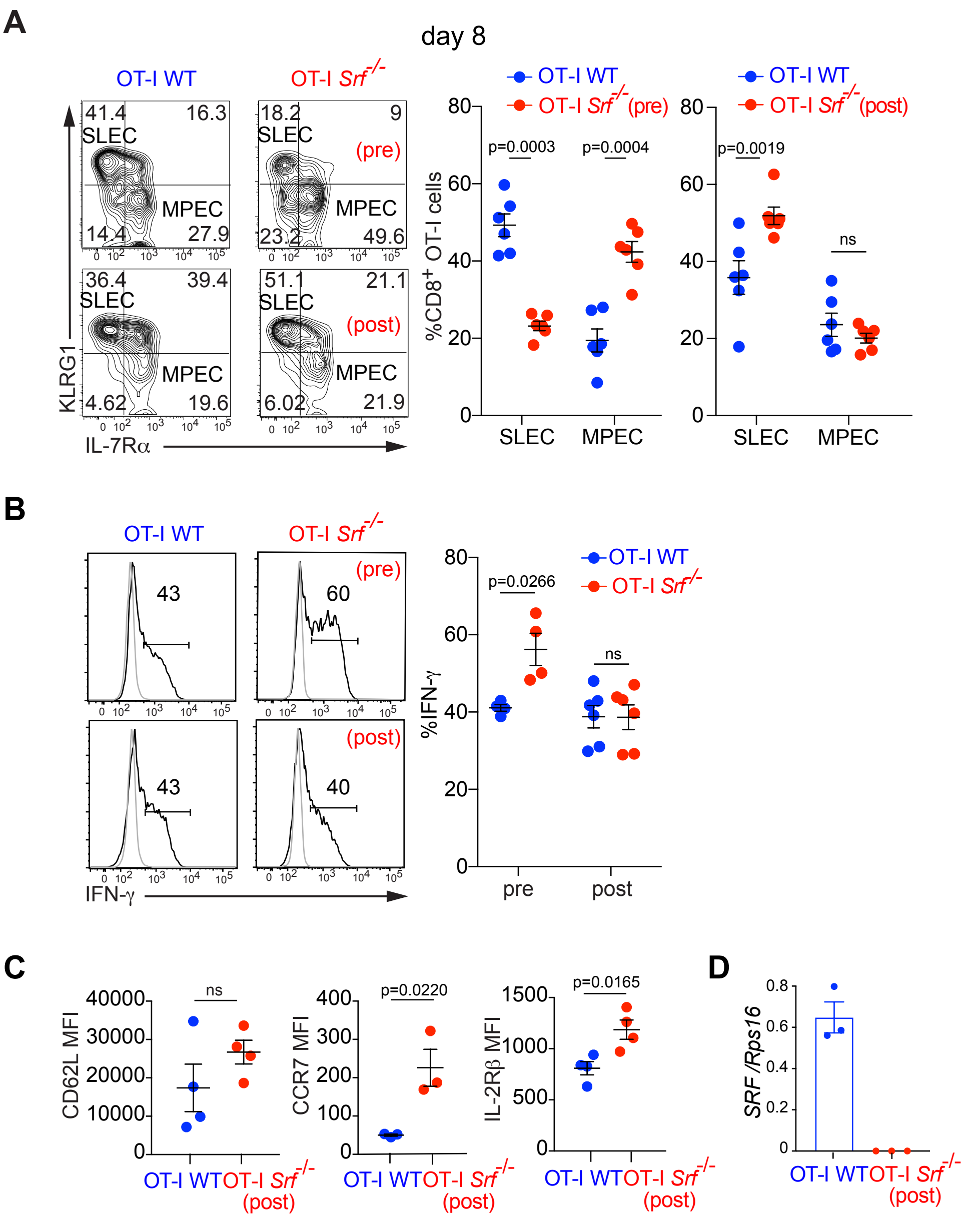

A
day 8
OT-I WT
OT-I Srf-/-
(pre)
(post)
SLEC
MPEC
KLRG1
IL-7Rα
41.4
16.3
14.4
27.9
18.2
9
23.2
49.6
36.4
39.4
4.62
19.6
51.1
21.1
6.02
21.9
%CD8+ OT-I cells
p=0.0003
p=0.0004
p=0.0019
ns
B
43
60
40
IFN-γ
%IFN-γ
p=0.0266
pre
post
C
CD62L MFI
CCR7 MFI
IL-2Rβ MFI
p=0.0220
p=0.0165
D
SRF /Rps16

Figure S2. *Srf* is required for effective SLEC accumulation.

(A) MPEC and SLEC generation in infected splenocytes. Wildtype cells were co-transferred to wildtype mice with cells in which *Srf* was inactivated either prior to transfer (OT-I $Srf^{-/-}$(pre)) or after transfer (OT-I $Srf^{-/-}$(post)). Mice were infected as outlined in Fig.1A (OT-I $Srf^{-/-}$(pre)), or Fig. 2A (OT-I $Srf^{-/-}$(post)). Data show mean values ± SEM; data points represent individual mice; n=6.

(B) Cytokine production by infected splenocytes stimulated with SIINFEKL peptide (OVA; 10nM) for 5 hours. Wildtype cells were co-transferred with cells in which *Srf* was inactivated either prior to transfer (OT-I $Srf^{-/-}$(pre)) or following transfer and infection (OT-I $Srf^{-/-}$(post)). Data show mean values ± SEM; data points represent individual mice.

(C) Memory-like OT-I WT and $Srf^{-/-}$(post) cells, defined by CD62L, CCR7 and IL-2Rβ expression.

(D) *Srf* mRNA levels in sorted OT-I splenocytes at day 50 post-infection.

Source data are provided as a Source Data file.

Figure S3

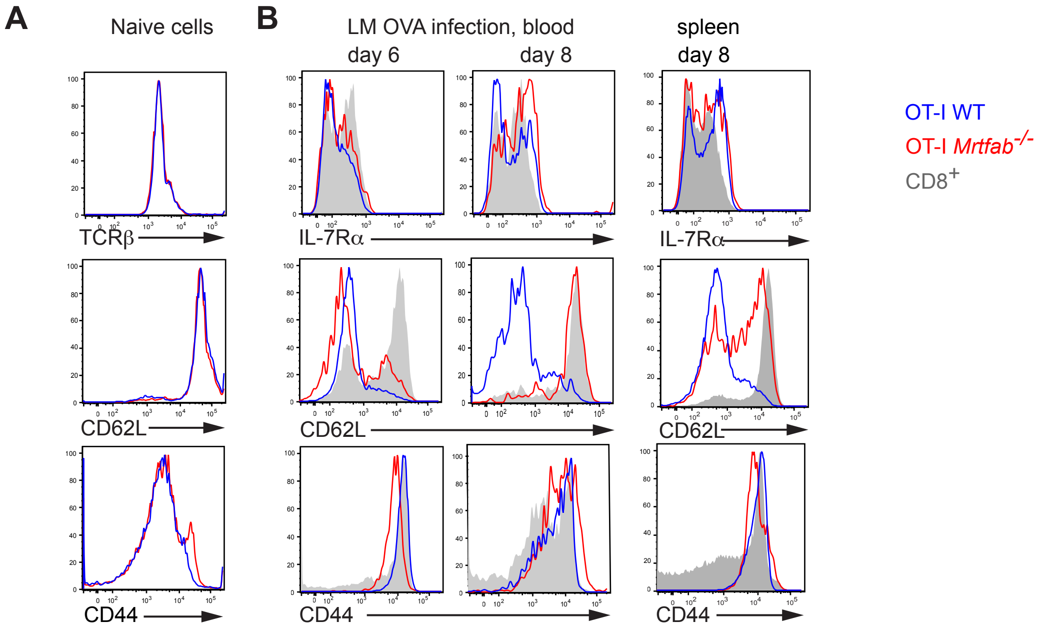

A
Naive cells
B
LM OVA infection, blood
day 6
day 8
spleen
day 8
TCRβ
IL-7Rα
CD62L
CD44
OT-I WT
OT-I Mrtfab-/-
CD8+

Figure S3. Phenotypic analysis of naïve OT-I *Mrtfab*$^{-/-}$ CD8^{+} T cells

(A) OT-I WT or OT-I *Mrtfab*$^{-/-}$ cells were MACS-purified from the spleens of tamoxifen-treated bone marrow reconstituted mice and stained for TCRβ, CD62L and CD44 expression.

(B) Expression of IL-7Rα CD62L and CD44 on OT-I WT or OT-I *Mrtfab*$^{-/-}$ CD8^{+} T cells following LM-OVA infection in blood and spleen.

Figure S4

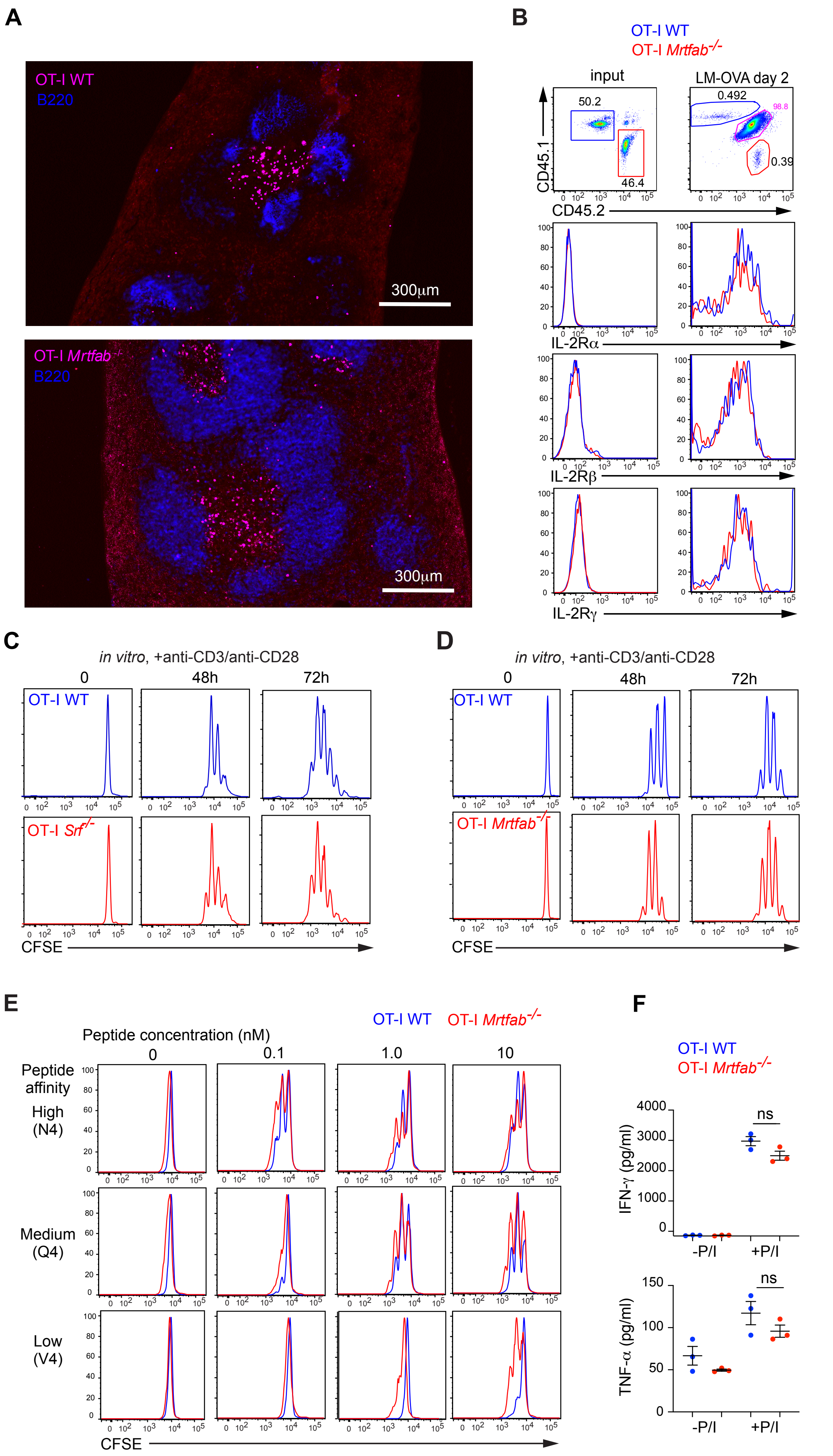
A
OT-I WT
B220
300µm
OT-I Mrtfab-/-
B220
300µm
B
OT-I WT
OT-I Mrtfab-/-
input
LM-OVA day 2
50.2
46.4
0.492
98.8
0.39
CD45.1
CD45.2
IL-2Rα
IL-2Rβ
IL-2Rγ
C
in vitro, +anti-CD3/anti-CD28
0
48h
72h
OT-I WT
OT-I Srf-/-
CFSE
D
in vitro, +anti-CD3/anti-CD28
0
48h
72h
OT-I WT
OT-I Mrtfab-/-
CFSE
E
OT-I WT
OT-I Mrtfab-/-
Peptide concentration (nM)
0
0.1
1.0
10
Peptide affinity
High (N4)
Medium (Q4)
Low (V4)
CFSE
F
OT-I WT
OT-I Mrtfab-/-
ns
IFN-γ (pg/ml)
-P/I
+P/I
ns
TNF-α (pg/ml)
-P/I
+P/I

Figure S4. OT-I *Mrtfab*$^{-/-}$ cell localisation after LM-OVA infection

(A) OT-I *Mrtfab*$^{-/-}$ and OT-I WT CD8^{+} T cells both localise to the periarteriolar lymphoid sheath. OCT-embedded sections of spleens from mice injected with OT-I *Mrtfab*$^{-/-}$ or OT-I WT CD8^{+} T cells labelled with cell-tracker deep red harvested 3d following LM-OVA infection, and counterstained for B220 to reveal B cell follicles. Scale bar 300μm.

(B) Expression of IL-2Rα, β and γ chains in OT-I WT and OT-I *Mrtfab*$^{-/-}$ cells prior to or 2 days after LM-OVA infection.

(C) Representative CFSE division profiles of MACS-purified OT-I WT or OT-I *Srf*$^{-/-}$ cells, either resting or activated with plate-bound anti-CD3/CD28 (5μg/ml) for the indicated times.

(D) Representative CFSE division profiles of MACS-purified OT-I WT or OT-I *Mrtfab*$^{-/-}$ cells, cultured as in (C).

(E) Representative CFSE division profiles of MACS-purified OT-I WT or OT-I *Mrtfab*$^{-/-}$ cells, activated for 48h by indicated concentrations of OVA peptides of decreasing affinity (N4 > Q4 > V4) for the OT-I TCR.

(F) ELISA analysis of IFN-γ and TNF-α secretion by purified OT-I cells stimulated with PDBu and Ionomycin (P/I) for 24h.

Source data are provided as a Source Data file.

Figure S5

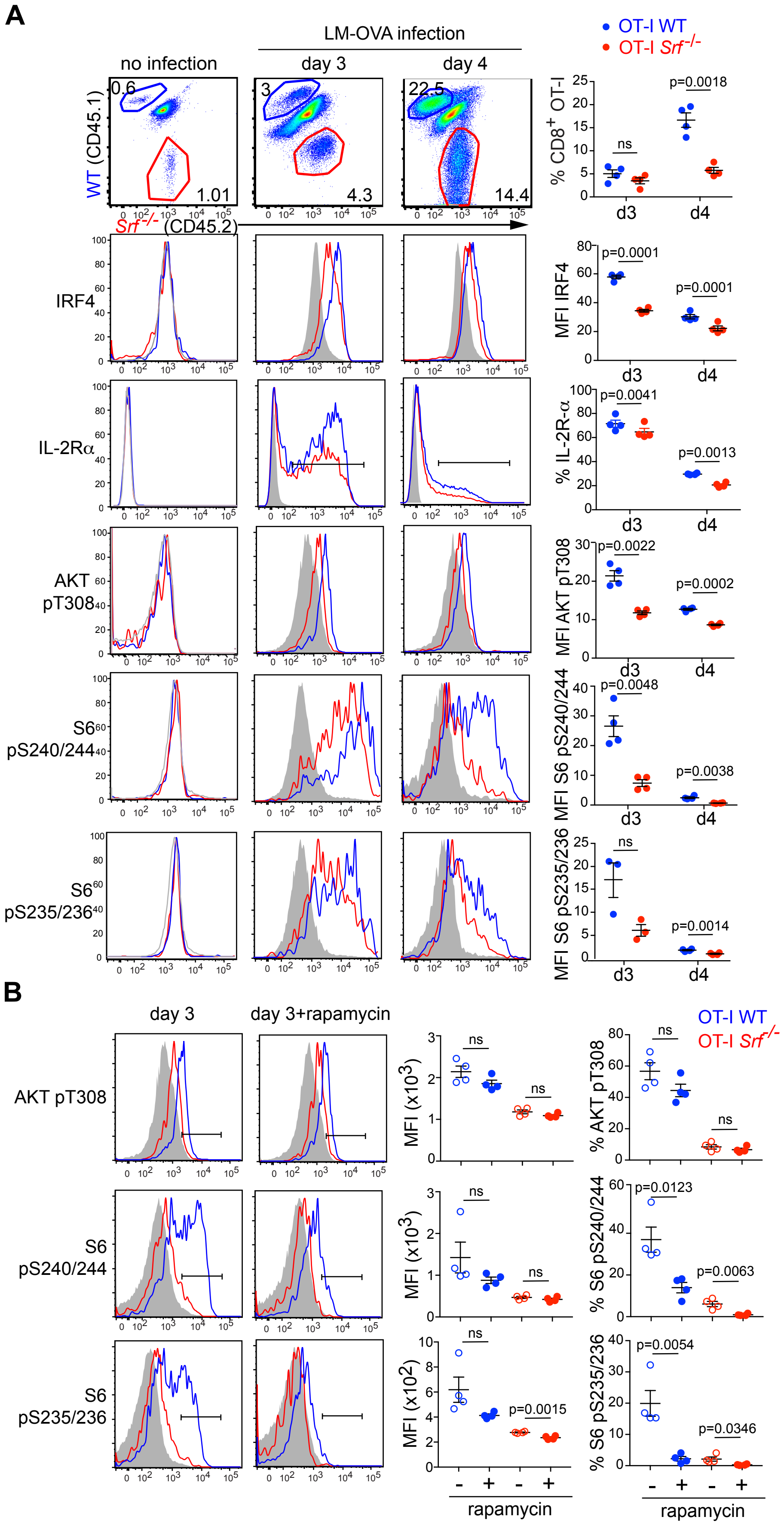

A
LM-OVA infection
no infection
day 3
day 4
OT-I WT
OT-I Srf-/-
WT (CD45.1)
Srf-/- (CD45.2)
0.6
1.01
3
4.3
22.5
14.4
% CD8+ OT-I
ns
p=0.0018
d3
d4
IRF4
MFI IRF4
p=0.0001
p=0.0001
IL-2Rα
% IL-2R-α
p=0.0041
p=0.0013
AKT pT308
MFI AKT pT308
p=0.0022
p=0.0002
S6 pS240/244
MFI S6 pS240/244
p=0.0048
p=0.0038
S6 pS235/236
MFI S6 pS235/236
ns
p=0.0014
B
day 3
day 3+rapamycin
OT-I WT
OT-I Srf-/-
AKT pT308
MFI (x10^3)
% AKT pT308
ns
S6 pS240/244
% S6 pS240/244
p=0.0123
p=0.0063
S6 pS235/236
MFI (x10^2)
% S6 pS235/236
p=0.0015
p=0.0054
p=0.0346
rapamycin

Figure S5. IL-2 signalling is defective in OT-I Srf$^{-/-}$ CD8^{+} T cells **during infection**

(A) Quantification of protein expression in OT-I CD8 splenocytes populations from mice co-transferred with OT-I WTCD45.1 and OT-I *Srf*$^{-/-}$ CD45.2 (1.10^{6} cells) and harvested at day 3 or 4 post-infection. A representative experiment is shown (at least 3 independent experiments per condition). Each data point represents a single mouse (n=4). Mean values ± SEM are shown, with statistical significance by paired t test.

(B) Expression of pAKT, pS6240/244 and 235/236 in OT-I WT (blue) and OT-I *Srf*$^{-/-}$ (red) and endogenous CD8^{+} T (grey-filled) cells in mice infected with LM-OVA. Splenocytes harvested 3 days after infection were treated or not with rapamycin (20nM) for 20 min *ex vivo* followed by antibody staining and analysis by flow cytometry. Statistical significance, paired t test.

Source data are provided as a Source Data file.

Figure S6

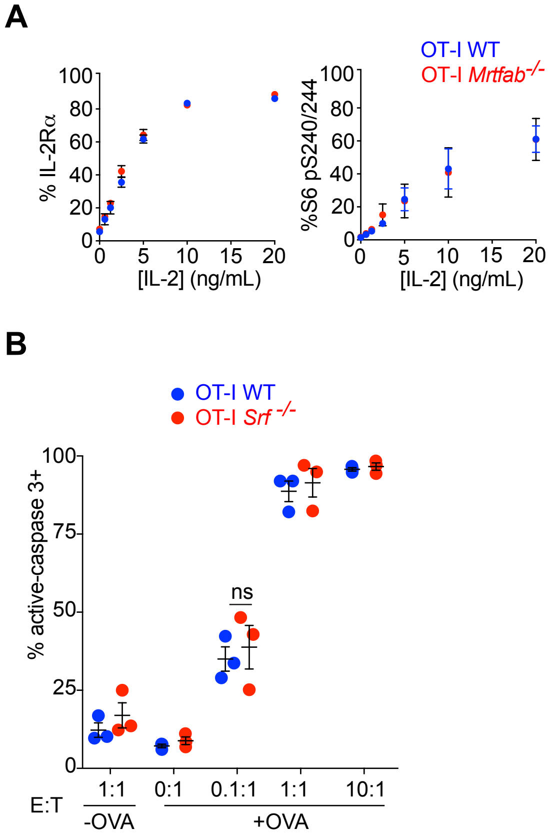

A
OT-I WT
OT-I Mrtfab-/-
% IL-2Rα
%S6 pS240/244
[IL-2] (ng/mL)
[IL-2] (ng/mL)
B
OT-I WT
OT-I Srf -/-
% active-caspase 3+
ns
E:T
1:1
0:1
0.1:1
1:1
10:1
-OVA
+OVA

Figure S6. Responses to exogenous IL-2 in *Srf*$^{-/-}$ and *Mrtfab*$^{-/-}$ cells

(A) OT-I lymph node cells were activated for 24h with plate-bound anti-CD3/CD28, washed and cultured for 24h with IL-2 then analysed for IL-2Rα (CD25) and S6 pS240/244.

(B) *In-vitro* killing by OT-I WT and OT-I *Srf*$^{-/-}$ effector CD8$^+$ T cells activated by plate-bound anti-CD3/CD28 and cultured for 7 days in IL-2 (20ng/ml), then incubated with OVA-pulsed target EL4 cells at different effector:target cell ratios. Killing of EL4 cells measured by intracellular caspase-3 staining. Data show mean values ± SEM; data points represent individual mice.

Source data are provided as a Source Data file.

Figure S7

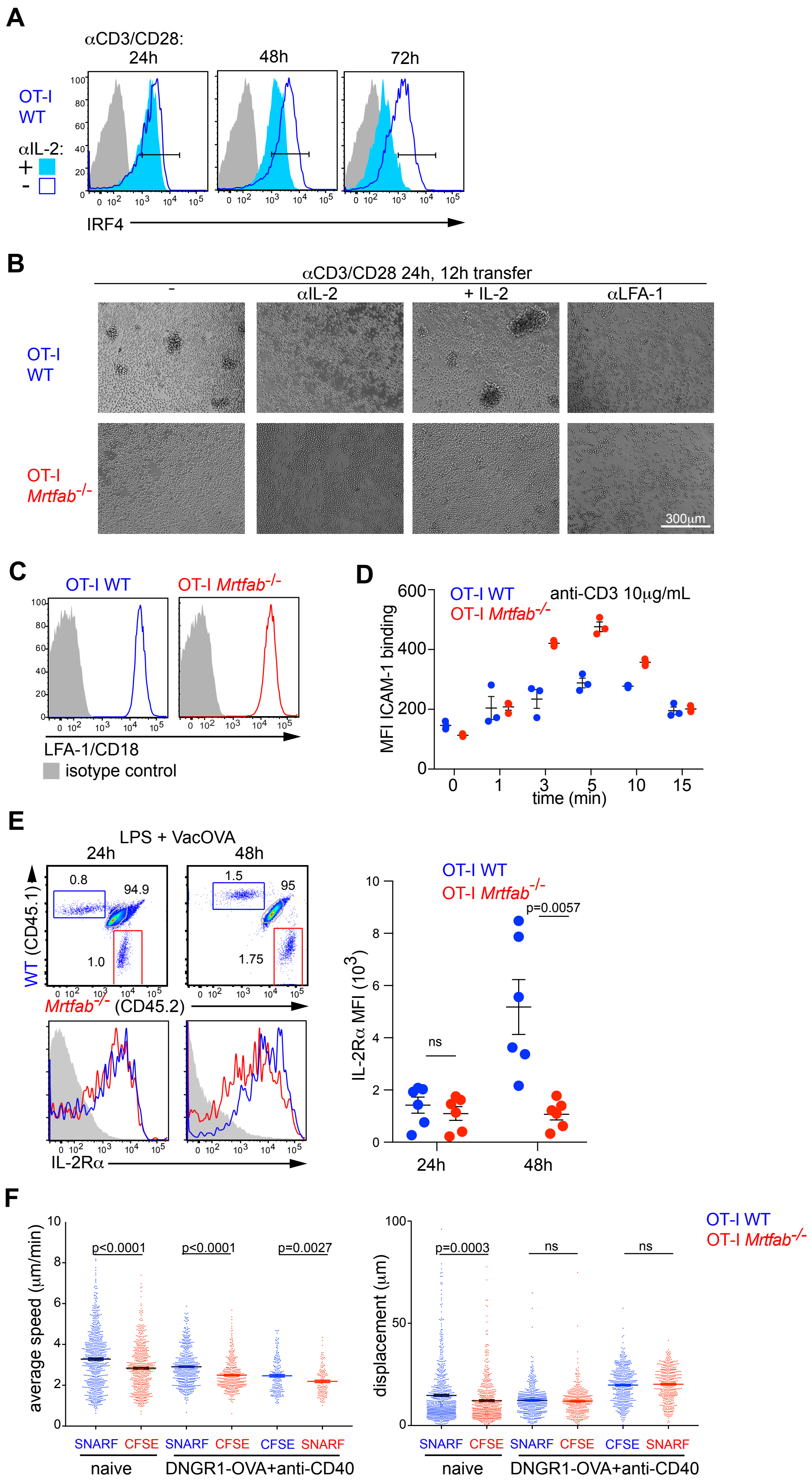
A
αCD3/CD28:
24h
48h
72h
OT-I
WT
αIL-2:
+
-
IRF4
B
αCD3/CD28 24h, 12h transfer
-
αIL-2
+ IL-2
αLFA-1
OT-I
WT
OT-I
Mrtfab-/-
300μm
C
OT-I WT
OT-I Mrtfab-/-
LFA-1/CD18
isotype control
D
OT-I WT
OT-I Mrtfab-/-
anti-CD3 10μg/mL
MFI ICAM-1 binding
time (min)
E
LPS + VacOVA
24h
48h
0.8
94.9
1.0
1.5
95
1.75
WT (CD45.1)
Mrtfab-/- (CD45.2)
IL-2Rα
OT-I WT
OT-I Mrtfab-/-
p=0.0057
ns
IL-2Rα MFI (10^3)
24h
48h
F
average speed (μm/min)
p<0.0001
p<0.0001
p=0.0027
SNARF CFSE SNARF CFSE CFSE SNARF
naive
DNGR1-OVA+anti-CD40
displacement (μm)
p=0.0003
ns
ns
SNARF CFSE SNARF CFSE CFSE SNARF
naive
DNGR1-OVA+anti-CD40
OT-I WT
OT-I Mrtfab-/-

Figure S7. Defective cluster formation by OT-I *Mrtfab*$^{-/-}$ cells

(A) Expression of IRF4 in MACS-purified OT-I WT and OT-I *Mrtfab*$^{-/-}$ isolated from lymph nodes from tamoxifen-fed bone marrow-reconstituted mice. Cells were activated *in vitro* with plate-bound anti-CD3/CD28 for the indicated times in the presence or absence of IL-2 blocking antibody.

(B) Cells were generated and activated as in (A), then transferred to fresh medium for 12h with IL-2 or IL-2 blocking antibody. Brightfield images of a representative experiment are shown (≥3 independent experiments). See also Fig.7C.

(C) Expression of LFA-1 in OT-I cells purified and activated as in (A).

(D) Time course of ICAM-1 binding to OT-I cells, purified as in (A), following activation by soluble anti-CD3.

(E) Wildtype mice were adoptively co-transferred with CD45.1 OT-I WT and CD45.2 OT-I *Mrtfab*$^{-/-}$ CD8^{+} T cells, immunised the following day, and IL-2Rα expression in inguinal lymph nodes analysed 24h and 48h later. IL-2Rα expression is quantified at right (OT-I WT (blue), OT-I *Mrtfab*$^{-/-}$ (red) endogenous control (grey)). Each data point represents an individual mouse, MFI quantified as mean value ± SEM, ** $p<0.01$, paired t test.

(F) Wild-type mice were co-injected with CFSE- or SNARF- labelled OT-I WT and OT-I *Mrtfab*$^{-/-}$ T cells and immunised with anti-DNGR1-OVA and anti-CD40, or PBS vehicle. Popliteal lymph nodes were removed 24h later and imaged by time-lapse microscopy. Data points are average speed or net displacement of individual cells over 15 min. Data are from 3 independent experiments.

Source data are provided as a Source Data file.

Figure S8

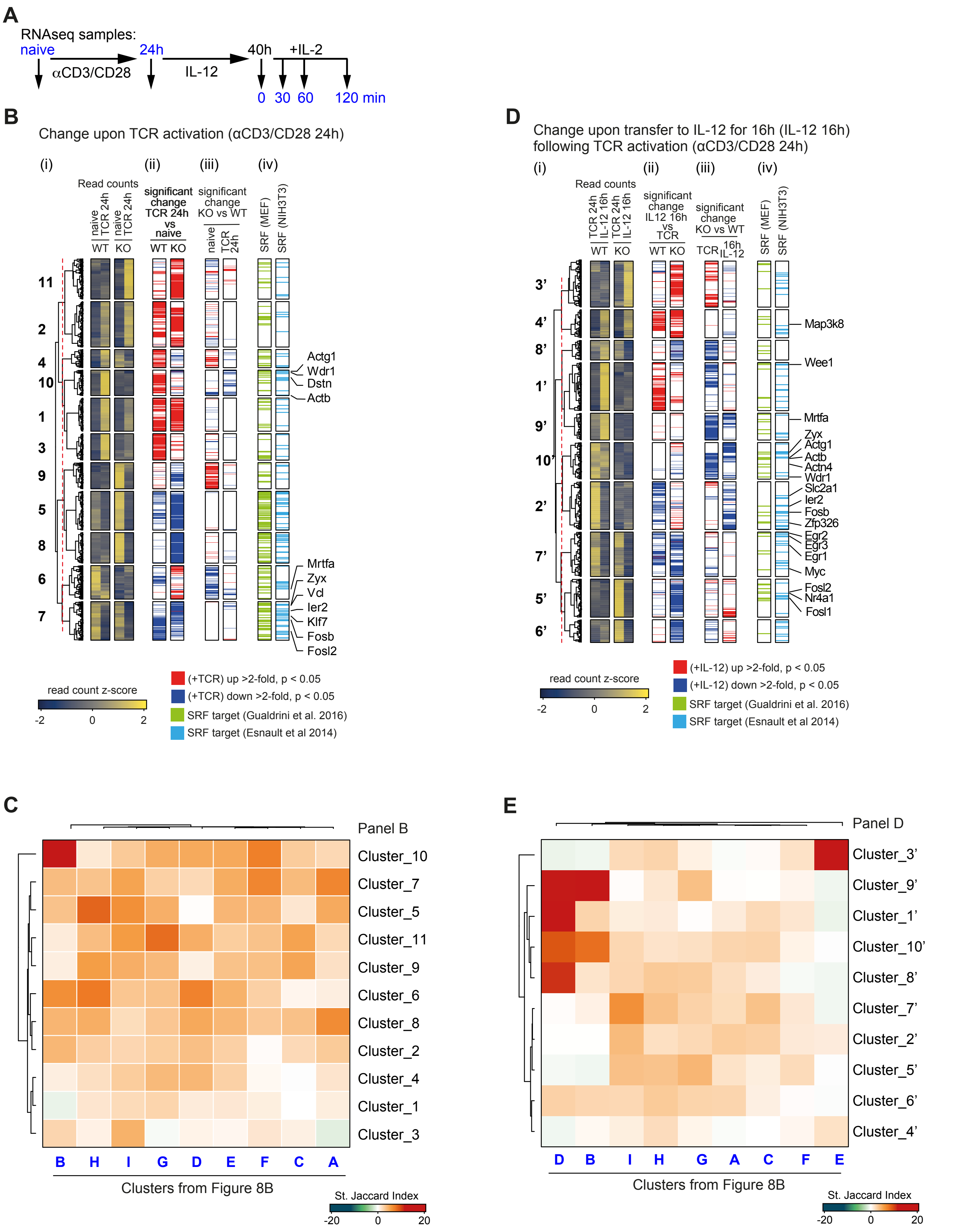
A
RNAseq samples:
naive
αCD3/CD28
24h
IL-12
40h
+IL-2
0 30 60 120 min
B
Change upon TCR activation (αCD3/CD28 24h)
(i) (ii) (iii) (iv)
Read counts
naive TCR 24h
naive TCR 24h
WT KO
significant change TCR 24h vs naive
WT KO
significant change KO vs WT
naive TCR 24h
SRF (MEF)
SRF (NIH3T3)
11
2
4
10
1
3
9
5
8
6
7
Actg1
Wdr1
Dstn
Actb
Mrtfa
Zyx
Vcl
Ier2
Klf7
Fosb
Fosl2
read count z-score
-2 0 2
(+TCR) up >2-fold, p < 0.05
(+TCR) down >2-fold, p < 0.05
SRF target (Gualdrini et al. 2016)
SRF target (Esnault et al 2014)
D
Change upon transfer to IL-12 for 16h (IL-12 16h)
following TCR activation (αCD3/CD28 24h)
(i) (ii) (iii) (iv)
Read counts
TCR 24h IL-12 16h
TCR 24h IL-12 16h
WT KO
significant change IL12 16h vs TCR
WT KO
significant change KO vs WT
TCR 16h IL-12
SRF (MEF)
SRF (NIH3T3)
3'
4'
8'
1'
9'
10'
2'
7'
5'
6'
Map3k8
Wee1
Mrtfa
Zyx
Actg1
Actb
Actn4
Wdr1
Slc2a1
Ier2
Fosb
Zfp326
Egr2
Egr3
Egr1
Myc
Fosl2
Nr4a1
Fosl1
read count z-score
-2 0 2
(+IL-12) up >2-fold, p < 0.05
(+IL-12) down >2-fold, p < 0.05
SRF target (Gualdrini et al. 2016)
SRF target (Esnault et al 2014)
C
Panel B
Cluster_10
Cluster_7
Cluster_5
Cluster_11
Cluster_9
Cluster_6
Cluster_8
Cluster_2
Cluster_4
Cluster_1
Cluster_3
B H I G D E F C A
Clusters from Figure 8B
St. Jaccard Index
-20 0 20
E
Panel D
Cluster_3'
Cluster_9'
Cluster_1'
Cluster_10'
Cluster_8'
Cluster_7'
Cluster_2'
Cluster_5'
Cluster_6'
Cluster_4'
D B I H G A C F E
Clusters from Figure 8B
St. Jaccard Index
-20 0 20

Figure S8. Gene expression deficits in OT-I *Mrtfab*$^{-/-}$ cells

(A) Stimulation protocol[53] for wildtype or *Mrtfab*-null OT-I CD8^{+} T cells.

(B) Unsupervised clustering was used to identify 11 groups of genes differentially responsive to 24h TCR activation. (i) Normalized z-scored read counts of genes differentially expressed in WT or *Mrtfab*-null cells following TCR activation for 24h, grouped by unsupervised clustering (clusters 1-11). Data show mean values ± SEM of three biological replicates. (ii) Genes showing significant changes upon TCR activation in wildtype (left) and *Mrtfab*-null cells (right). Genes showing an absolute fold change greater than 2 at p<0.05 (DESeq2) are colour coded. Fold-change and padj values are reported in Supplementary Data 1. (iii) Genes whose expression is impaired by MRTF inactivation in naïve cells (left) or TCR-activated cells (right), displayed as in (ii). (iv) Genes identified as candidate SRF targets in TPA-stimulated MEFs[42] or serum-stimulated NIH3T3 fibroblasts[41].

(C) Overlap testing analysis, displayed as standardised Jaccard score, of the relation between gene groups differentially expressed upon TCR-activation of WT or *Mrtfab*-null cells (clusters 1-11 in panel B) and those differentially expressed between WT and *Mrtfab*-null TCR-activated/rested cells with or without stimulation by IL-2 (see also Fig.8B, groups A-I). St.JI scores and Adjp values are reported in Supplementary Data 2.

(D) Unsupervised clustering was used to identify groups of genes differentially responsive to activation and subsequent resting in IL-12 for 16h. (i) Normalized z-scored read counts of genes differentially expressed in WT or *Mrtfab*-null cells upon resting in IL-12 following TCR activation (clusters 1'-10'). Data show mean values ± SEM of three biological replicates. (ii) Genes showing significant changes following culture in IL-12 in wildtype (left) and *Mrtfab*-null cells (right). Genes showing an absolute fold change greater than 2 at p<0.05 (DESeq2) are colour-coded. Fold-change and padj values are reported in Supplementary Data 1. (iii) Genes whose expression is impaired by MRTF inactivation, in TCR-activated cells (left) or TCR-activated/rested cells (right), displayed as in (ii). (iv) Genes identified as candidate SRF targets in TPA-stimulated MEFs[42] or serum-stimulated NIH3T3 fibroblasts[41].

(E) Overlap testing analysis, displayed as standardised Jaccard score, of the relation between gene groups differentially expressed in WT or *Mrtfab*-null cells upon resting in IL-12 following TCR activation (clusters 1'-10' in panel D) and those differentially expressed between WT and *Mrtfab*-null TCR-activated/rested cells (see also Fig.8B, groups A-I). St.JI scores and Adjp values are reported in Supplementary Data 2.

Figure S9

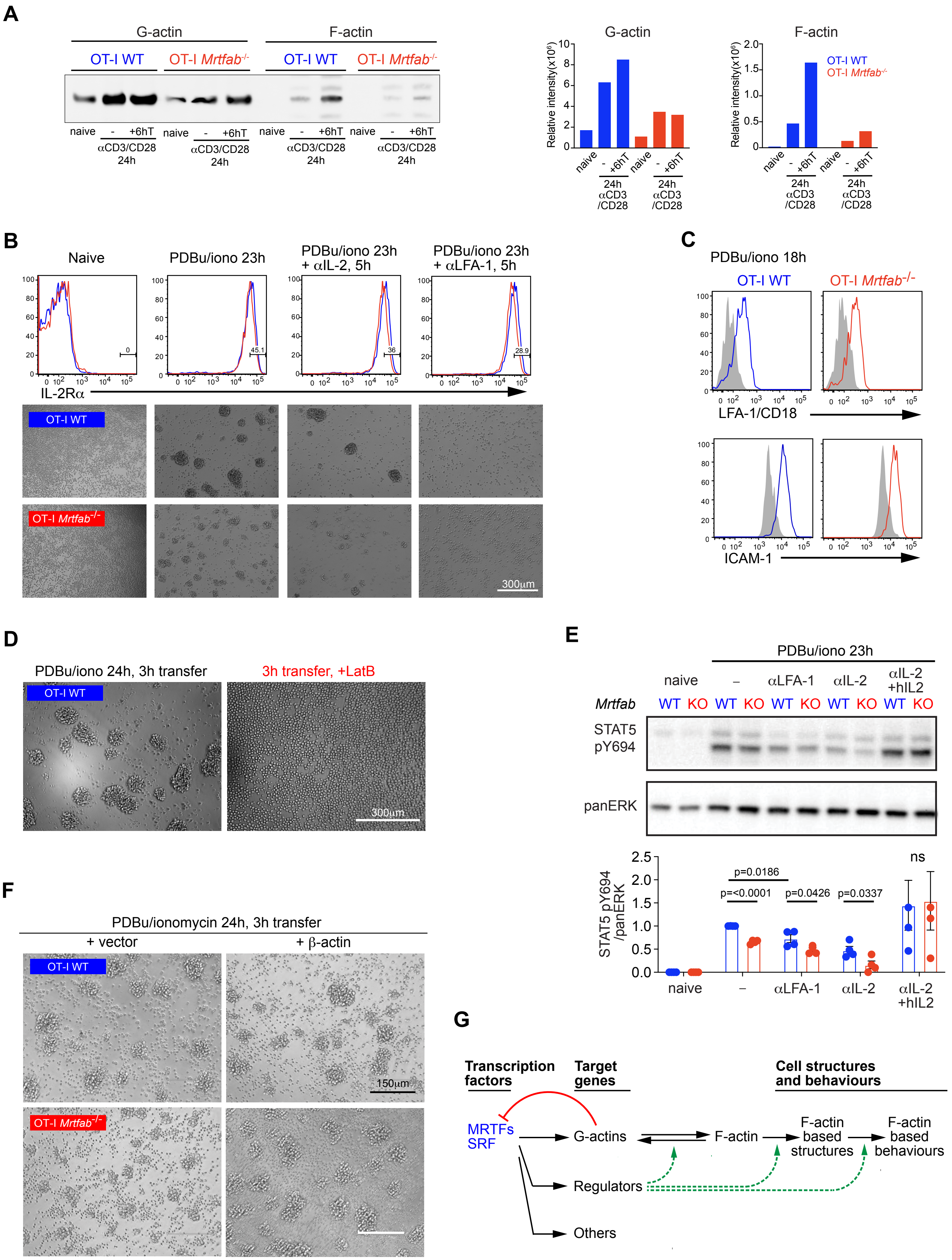

A
G-actin
F-actin
OT-I WT
OT-I Mrtfab-/-
naive
-
+6hT
αCD3/CD28
24h
Relative intensity(x10⁶)
OT-I WT
OT-I Mrtfab-/-
24h
αCD3
/CD28
B
Naive
PDBu/iono 23h
PDBu/iono 23h
+ αIL-2, 5h
PDBu/iono 23h
+ αLFA-1, 5h
IL-2Rα
OT-I WT
OT-I Mrtfab-/-
300μm
C
PDBu/iono 18h
OT-I WT
OT-I Mrtfab-/-
LFA-1/CD18
ICAM-1
D
PDBu/iono 24h, 3h transfer
3h transfer, +LatB
OT-I WT
300μm
E
PDBu/iono 23h
naive
–
αLFA-1
αIL-2
αIL-2
+hIL2
Mrtfab
WT KO WT KO WT KO WT KO WT KO
STAT5
pY694
panERK
STAT5 pY694
/panERK
p=0.0186
p=<0.0001
p=0.0426
p=0.0337
ns
F
PDBu/ionomycin 24h, 3h transfer
+ vector
+ β-actin
OT-I WT
OT-I Mrtfab-/-
150μm
G
Transcription
factors
Target
genes
Cell structures
and behaviours
MRTFs
SRF
G-actins
F-actin
F-actin
based
structures
F-actin
based
behaviours
Regulators
Others

Figure S9. MRTF-dependent F-actin assembly in homotypic clustering

(A) Cluster formation requires F-actin. OT-I WT CD8$^+$ T cells were activated *in vitro* for 24h on plate-bound anti-CD3/CD28, then transferred to uncoated wells for 6h. Actin in the supernatant and pellet fractions from the F-actin sedimentation assay was analysed by immunoblotting (see also Fig.9C). A representative of two independent experiments is shown.

(B) MACS-purified OT-I CD8+ splenocytes were stimulated with PDBu and Ionomcyin for 18h to induce cluster formation, then treated with anti-LFA-1 or anti-IL-2 blocking antibody for 5h. Top, IL-2Rα expression assessed by flow cytometry. Bottom, brightfield images of cluster formation.

(C) LFA-1 and ICAM expression in cells stimulated as in (B).

(D) OT-I WT CD8$^+$ T cells were activated as in (B) then transferred to fresh culture for 3h with or without 5μM LatB. Representative brightfield images are shown (2 independent experiments)

(E) STAT5 pY694 expression following cluster induction and treatment as in (B). Bottom, quantitation, with normalisation to level in wildtype activated cells (± SEM, n= 3).

(F) Brightfield images (20x magnification) of PDBu/ionomycin induced clusters in WT and OT-I cells infected with either lentiviral vectors expressing either mCherry alone or mCherry-β-actin (see also Fig.9G).

(G) MRTF-SRF transcriptional targets and cytoskeletal regulatory interactions. Red inhibitory arrow, G-actin control of MRTF activity constitutes a homeostatic feedback loop[61,39]. Green positive arrows indicate MRTF-SRF control of positive regulators of F-actin assembly and treadmilling, F-actin crosslinking, and actin-based cell behaviours such as contractility, motility and adhesion[41]. At least in some contexts, actin expression can suppress MRTF-null phenotypes associated with MRTF inactivation (see Ref [43] and Fig.9G).

Source data are provided as a Source Data file.

Figure S10

A

Live cells

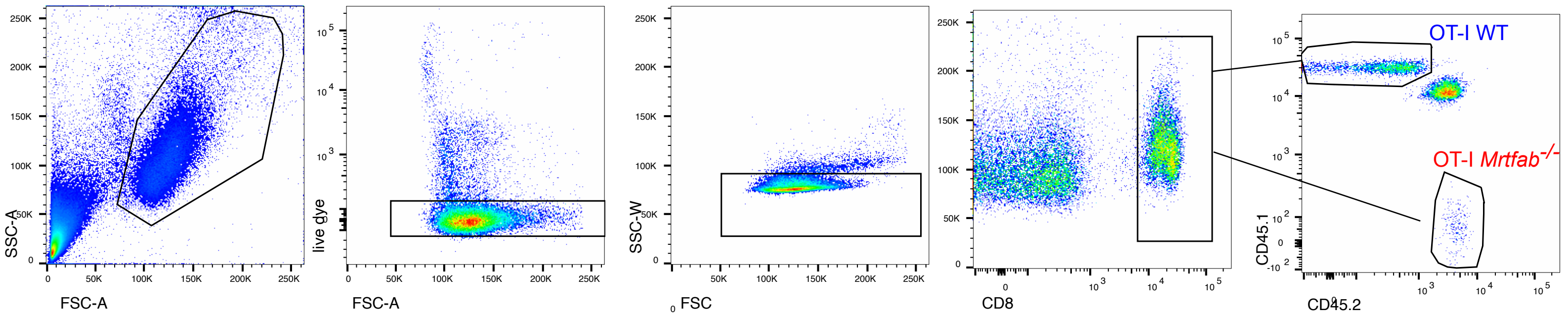

B

Fixed cells

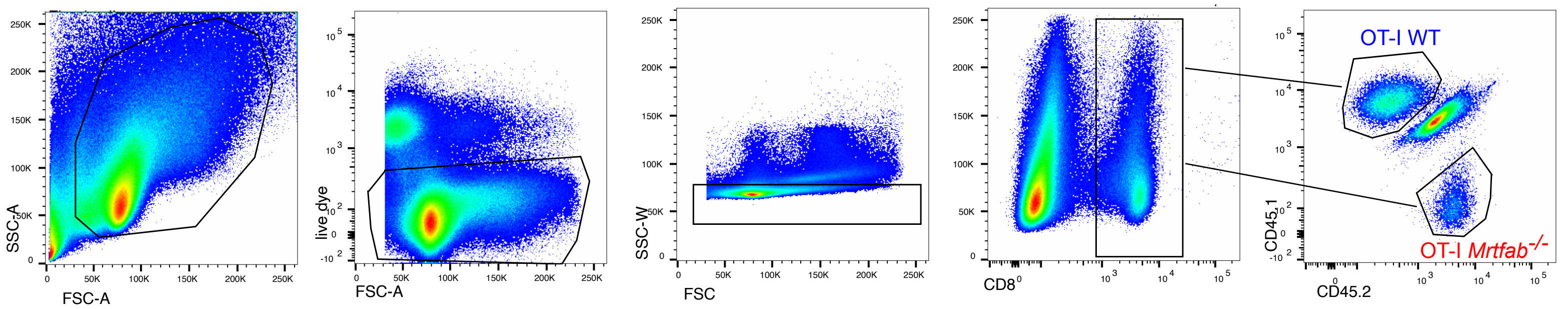

C

Sort

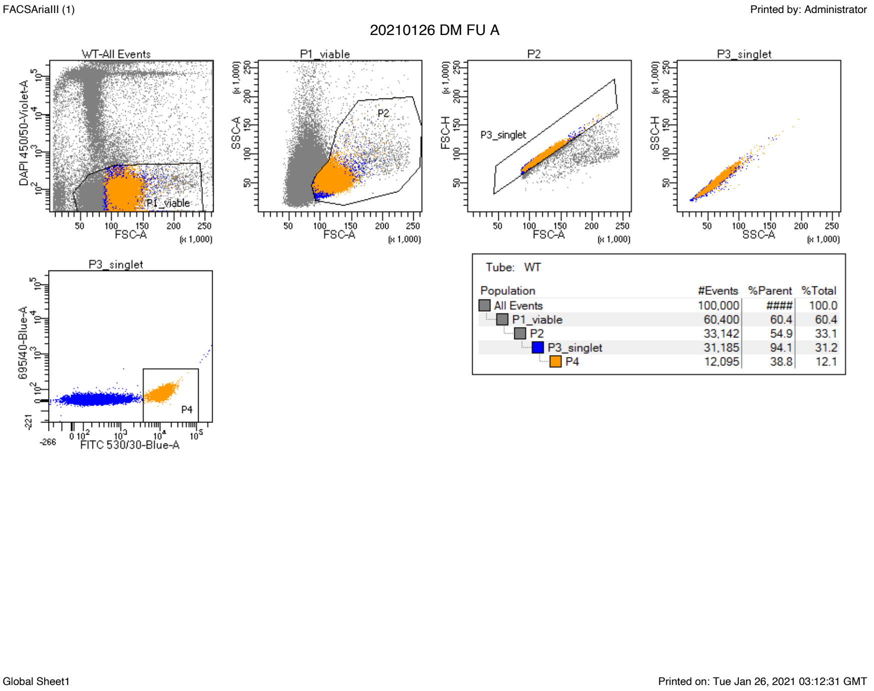

Figure S10. FACS gating and sorting strategies.

(A) and **(B)**. Gating strategy used to display both extracellular and intracellular proteins expressed by co-transferred OTI-WT (CD45.1) and OTI-KO (CD45.2) CD8 T cells in mice infected with Listeria-OVA. This gating strategy is used in Fig1C,1D,1E, 2B, 2E, 2F, 3B, 3F, 3G, 4A, 4B, 4C, 5A, 5B, 6A and 6B.

(C) Gating strategy to sort OT-I WT and OT-I *Mrtfab*$^{-/-}$ CD8+ T cells from lymph nodes of bone marrow reconstituted mice for RNA seq Fig 8A,8B,8C and 8D.

Supplementary Data 1. Gene expression in OT-I CD8$^+$ T cells

1. Summary

Structure of Sheets 2, 3,4

2. Differential Gene Expression

This reports the behaviour of the 5659 genes identified as differentially expressed in at least one of the comparisons: Naive to TCR 24h transition (Figure S8B); TCR 24h to rested IL-12 transition (Figure S8C) and the IL-2 time-course (Figure 8B). Z scores are shaded from positive (dark red) to negative (dark blue) values. #N/A, not called as differentially expressed in that comparison.

Genes are listed according to the clusters defined by differential response to IL-2 (Figure 8B), allowing comparison with responses to TCR activation (Figure S8B) and/or resting of activated cells in IL-12 for 12h (Figure S8C).

A	Gene identifier
B-F	Naïve to TCR 24h transition
B-E	Z scores for individual genes during TCR activation as in Figure S8B. B-C, WT cells, D-E, KO cells.
F	Lists which cluster these fall into in the analysis shown in Figure S8B.
G-K	TCR 24h to rested in IL-12 transition
G-J	Z scores for individual genes during upon resting of TCR-activated cells in IL-12 transition as in Figure S8C. G-H, WT cells, I-J, KO cells.
K	Lists which cluster these fall into in the analysis shown in Figure S8C.
L-T	IL-2 time course
L-O, P-S	Z scores for individual genes in activated/rested cells after 0, 30, 60 or 120 minutes of IL-2 stimulation. L-O, WT cells, P-S, KO cells.
T	Lists which cluster these fall into as in Figure 8B.
U	Whether gene concerned is a TCF direct target[42]
V	Whether gene concerned is an Actin cytoskeleton gene as defined by MsigDB: https://www.gsea-msigdb.org/gsea/msigdb/mouse/geneset/GOCC_ACTIN_CYTOSKELETON.html

3. All Reads Analysis

4. Intronic Reads Analysis

These tables summarise gene expression under different conditions in WT or *mrtfab*$^{-/-}$ OT-1 CD8^{+} T cells, measured by total read-counts (sheet 3) or intronic read-counts (sheet 4) per kb.

A: Gene index

B : Gene name

C: TCF direct target[42]

D: Actin cytoskeleton genes from MsigDB:

https://www.gsea-msigdb.org/gsea/msigdb/mouse/geneset/GOCC_ACTIN_CYTOSKELETON.html

E-J: read-counts in WT cells - resting, TCR24h, activated/rested, +IL2 30' 60' 120'

K-P: read-counts in KO cells - resting, TCR24h, activated/rested, +IL2 30' 60' 120'

Q-T, U-X,Y-AB, AC-AF, AG-AJ,AK-AN, AO-AR, AS-AV, AW-AZ, BA-BD,BE-BH, BI-BL, BM-BP, BQ-BT, BU-BX, and BY-CB: each of these groups of four columns summarises the results of the individual comparisons. Each comparison is identified in row 1, along with the total number of genes showing a >2 fold increase or decrease for the comparison. For each group, the first two columns show the log2 fold-change and padj, as determined by DESeq2 (See Methods), and the second two score scenes showing >2 fold increase or decreased expression at $p<0.05$.

Supplementary Data 2. Gene cluster analysis

The enrichment of gene categories for either Reactome or Gene Ontology sets from the MSigDB was computed for gene clusters A-I (Figure 8B), 1-11 (Figure S8B), and 1'-10' (Figure S8D). This analysis utilized the Standardized Jaccard Index in conjunction with hypergeometric testing following multiple test correction.

Within each Sheet column A gives the GO or Reactome ID. Subsequent columns summarise for each cluster the St.JI, hypergeometric adjpval and the genes in common between the cluster and each MSigDB gene list. St.JI values are shaded from positive (dark red) to negative (dark blue).

Sheets are as follows:

1. Legend
2. GO IL-2_TC_Clusters: Gene Ontology enrichment for clusters A-I, Figure 8B. Default sort order gives categories for Figure 8B, cluster B genes, sorted by increasing hypogeometric adjpval.
3. REACTOME IL-2_TC_Clusters: Reactome gene class enrichment for clusters A-I, Figure 8B. Default sort order gives categories for Figure 8B, cluster B genes, cluster 10 genes, sorted by increasing hypogeometric adjpval.
4. GO NAIVE to TCR 24h: Gene Ontology enrichment for the clusters in Figure S8B. Default sort order gives categories for Figure S8B, cluster 10 genes, sorted by increasing hypogeometric adjpval.
5. REACTOME NAIVE to TCR 24h: Reactome gene class enrichment for the clusters in Figure S8B. Default sort order gives categories for Figure S8B, cluster 10 genes, sorted by increasing hypogeometric adjpval.
6. GO TCR 24h to resting IL-12: Gene Ontology enrichment for the clusters in Figure S8D. Default sort order gives categories for Figure S8D, cluster 10' genes, sorted by increasing hypogeometric adjpval.
7. REACTOME TCR 24h to resting IL-12: Reactome gene class enrichment for the clusters in Figure S8D. Default sort order gives categories for Figure S8D, cluster 10' genes, sorted by increasing hypogeometric adjpval.
