# Supplementary material for: IL-2 delivery to CD8+ T cells during infection requires MRTF/SRF-dependent gene expression and cytoskeletal dynamics
Source: Nat Commun. 2024 Sep 11;15:7956. doi: 10.1038/s41467-024-52230-8 (PMC11391060; doi:10.1038/s41467-024-52230-8)
Supplement: Supplementary file 3 — Description of Additional Supplementary Files [file 41467_2024_52230_MOESM3_ESM.pdf]

Description of Additional Supplementary Files

File Name: Supplementary Data 1
Description: Gene expression in $CD8^+$ T cells

File Name: Supplementary Data 2
Description: Gene cluster analysis
