# Supplementary material for: IL-2 delivery to CD8+ T cells during infection requires MRTF/SRF-dependent gene expression and cytoskeletal dynamics
Source: Nat Commun. 2024 Sep 11;15:7956. doi: 10.1038/s41467-024-52230-8 (PMC11391060; doi:10.1038/s41467-024-52230-8)
Supplement: Supplementary file 6 — Reporting Summary [file 41467_2024_52230_MOESM6_ESM.pdf]

nature portfolio

Corresponding author(s): Richard Treisman

Last updated by author(s): 12 August 2024

Reporting Summary

Nature Portfolio wishes to improve the reproducibility of the work that we publish. This form provides structure for consistency and transparency in reporting. For further information on Nature Portfolio policies, see our Editorial Policies and the Editorial Policy Checklist.

Statistics

For all statistical analyses, confirm that the following items are present in the figure legend, table legend, main text, or Methods section.

n/a	Confirmed	
☐	☒	The exact sample size (*n*) for each experimental group/condition, given as a discrete number and unit of measurement
☐	☒	A statement on whether measurements were taken from distinct samples or whether the same sample was measured repeatedly
☐	☒	The statistical test(s) used AND whether they are one- or two-sided<br>*Only common tests should be described solely by name; describe more complex techniques in the Methods section.*
☐	☒	A description of all covariates tested
☐	☒	A description of any assumptions or corrections, such as tests of normality and adjustment for multiple comparisons
☒	☐	A full description of the statistical parameters including central tendency (e.g. means) or other basic estimates (e.g. regression coefficient) AND variation (e.g. standard deviation) or associated estimates of uncertainty (e.g. confidence intervals)
☐	☒	For null hypothesis testing, the test statistic (e.g. *F*, *t*, *r*) with confidence intervals, effect sizes, degrees of freedom and *P* value noted<br>*Give P values as exact values whenever suitable.*
☒	☐	For Bayesian analysis, information on the choice of priors and Markov chain Monte Carlo settings
☒	☐	For hierarchical and complex designs, identification of the appropriate level for tests and full reporting of outcomes
☒	☐	Estimates of effect sizes (e.g. Cohen's *d*, Pearson's *r*), indicating how they were calculated

Our web collection on statistics for biologists contains articles on many of the points above.

Software and code

Policy information about availability of computer code

Data collection	BD INFLUX or FACS ARIA-III, ZEISS Observer D1 AxioCam Mrm, Zeiss LSM710 invert, Zeiss LSM880 invert with an airyscan module, LSM Zeiss 710 multiphoton
Data analysis	BD FACS Diva software, Graph Pad Prism 6, Imaris 9.5 and 9.6 , CellProfiler v4.2.4, FIJI 10 , Bioinformatics analysis methods and code are available at https://github.com/fgualdr?tab=repositories

For manuscripts utilizing custom algorithms or software that are central to the research but not yet described in published literature, software must be made available to editors and reviewers. We strongly encourage code deposition in a community repository (e.g. GitHub). See the Nature Portfolio guidelines for submitting code & software for further information.

Data

Policy information about availability of data

All manuscripts must include a data availability statement. This statement should provide the following information, where applicable:

- Accession codes, unique identifiers, or web links for publicly available datasets
- A description of any restrictions on data availability
- For clinical datasets or third party data, please ensure that the statement adheres to our policy

GEO number: GSE241689

Research involving human participants, their data, or biological material

Policy information about studies with human participants or human data. See also policy information about sex, gender (identity/presentation), and sexual orientation and race, ethnicity and racism.

Reporting on sex and gender	*Use the terms sex (biological attribute) and gender (shaped by social and cultural circumstances) carefully in order to avoid confusing both terms. Indicate if findings apply to only one sex or gender; describe whether sex and gender were considered in study design; whether sex and/or gender was determined based on self-reporting or assigned and methods used. Provide in the source data disaggregated sex and gender data, where this information has been collected, and if consent has been obtained for sharing of individual-level data; provide overall numbers in this Reporting Summary. Please state if this information has not been collected. Report sex- and gender-based analyses where performed, justify reasons for lack of sex- and gender-based analysis.*
Reporting on race, ethnicity, or other socially relevant groupings	*Please specify the socially constructed or socially relevant categorization variable(s) used in your manuscript and explain why they were used. Please note that such variables should not be used as proxies for other socially constructed/relevant variables (for example, race or ethnicity should not be used as a proxy for socioeconomic status). Provide clear definitions of the relevant terms used, how they were provided (by the participants/respondents, the researchers, or third parties), and the method(s) used to classify people into the different categories (e.g. self-report, census or administrative data, social media data, etc.) Please provide details about how you controlled for confounding variables in your analyses.*
Population characteristics	*Describe the covariate-relevant population characteristics of the human research participants (e.g. age, genotypic information, past and current diagnosis and treatment categories). If you filled out the behavioural & social sciences study design questions and have nothing to add here, write "See above."*
Recruitment	*Describe how participants were recruited. Outline any potential self-selection bias or other biases that may be present and how these are likely to impact results.*
Ethics oversight	*Identify the organization(s) that approved the study protocol.*

Note that full information on the approval of the study protocol must also be provided in the manuscript.

Field-specific reporting

Please select the one below that is the best fit for your research. If you are not sure, read the appropriate sections before making your selection.

☒ Life sciences ☐ Behavioural & social sciences ☐ Ecological, evolutionary & environmental sciences

For a reference copy of the document with all sections, see nature.com/documents/nr-reporting-summary-flat.pdf

Life sciences study design

All studies must disclose on these points even when the disclosure is negative.

Sample size	SRF and MRTF mice studies: at least 6-8 reconstituted mice were analysed for each experiment. Reconstituted mice consist of both WT and KO CD8 T cells. These experiments include kinetic of activation and differentiation following Listeria-OVA infection and cell signalling. The numbers of mice used was proportional to the effects measured in the preliminary experiments and in agreement with the 3Rs.These experiments were replicated in both the SRFKO background and the MRTFAB KO background with similar results suggesting that sample size was adequate.<br>In vitro activation experiments were performed with at least three independent biological replicates and generally done twice.<br>Imaging experiments: in vitro generated clusters; 7-8 clusters/genotype were imaged and characterised for cell numbers, distance between cells, phalloidin content, sphericity and IL-2 retention. Results show statistical significant differences between WT and MRTFABKO genotypes suggesting adequate sample size.<br>In vivo imaging: 25 images of lymph nodes containing both activated WT and MRTFAB KO CD8 T cells in similar numbers were analysed for presence of clusters and compositions of contacts. Statistical analysis show more than 2 fold defects in the ability of KO cells to establish contact by comparison to WT suggesting adequate sample sizing.<br>Live imaging: Data pooled from 3 independent experiments a , tracking both WT and MRTFAB KO cells coinjected in the same popliteal lymph nodes. Each individual cell is tracked over 15 minutes.<br>RNA seq was performed on 3 biological replicates for each genotype (Bioinformatics analysis methods and code are available at https://github.com/fgualdr?tab=repositories)
Data exclusions	No individual data points were excluded from the analysis under any circumstances
Replication	All experiments were replicated appropriately (at least twice) and only data that reproduced across all experiments were included in this manuscript.
Randomization	Mice were not randomized in cages but each cage contained both control and experimental mice submitted to identical treatment.
Blinding	Investigators were not blinded to mice identity during sample analysis.

Reporting for specific materials, systems and methods

We require information from authors about some types of materials, experimental systems and methods used in many studies. Here, indicate whether each material, system or method listed is relevant to your study. If you are not sure if a list item applies to your research, read the appropriate section before selecting a response.

Materials & experimental systems

n/a	Involved in the study
☐	☒ Antibodies
☒	☐ Eukaryotic cell lines
☒	☐ Palaeontology and archaeology
☐	☒ Animals and other organisms
☒	☐ Clinical data
☒	☐ Dual use research of concern
☒	☐ Plants

Methods

n/a	Involved in the study
☒	☐ ChIP-seq
☐	☒ Flow cytometry
☒	☐ MRI-based neuroimaging

Antibodies

Antibodies used

Antibodies for extracellular markers were used at a dilution of 1:200 and purchased from: eBioscience: CD8 (53-6.7), CD122 (5H4), TCRb (H57-597), CD44 (IM7), CD5 (53-7.3), CD62L (MEL-14), CCR7 (4B12), NKG2D (CX5), LFA-1/CD11a (M17/4). BD biosciences: CD11b (M1/70), CD45.1 (A20), CD45.2 (104), CD4 (RM4-5), CD127 (SB/199), KLRG1 (2F1), TCRb (H57-597), CD132 (TUGm2), CD25 (PC61), CD69 (H1.2F3). Biolegend: CD11a/CD18 (H155-78), CD62L(MEL-14), CD69 (H1.2F3), CCL5 (2E9), B220 (RA3-GB2). Streptavidin Brilliant violet 421.Jackson Immunoresearch: APC goat anti-human IgG F(ab')2 fragments (109-135-098), R&D systems: rmICAM1-Fc (796-IC-050).
Antibodies for intracellular staining were used at a dilution of 1:200 unless stated otherwise, and purchased from: eBioscience: IRF4 (3E4), IFNg (XMG1.2), GranzymeB (NGZB), Fixable viability dye eFluor780 1:500 (65-0865-14). BD Biosciences: Bcl-2 (3F11), Caspase-3 (C92-605), pSTAT5(pY694) (47) (562077), panSTAT5 (89), (610192), TNF (MP6-XT22), BrdU FITC 1:50 (556028), CD16/32 FcBlock 1:100. Cell Signaling: pAKT(T308) (D25E6), pS6 240/244 (D68F8), pS6 235/236 (D57.2.2E), p44/42-ERK (D13.14.4E), pStat5 (pY694) (9351), Stat5 (D206Y) (94205).
Invitrogen: anti-DNaseI 488 1:200 (D12371 5mg/ml),Phalloidin Alexa Fluor 647 1:50 (A22287), CellTrace CFSE 4uM, SNARFTM 2.8uM.
Antibodies used for Western blotting were used at 1:1000 unless stated otherwise and purchased from: Santa-Cruz Biotechnology: anti-SRF (G20). BD Biosciences: panERK(16), panSTAT5 (89). Bethyl laboratories: MRTF-B (A302-786A). Biolegend: GAPDH 1/5000 W17079A (607901). Cell Signaling: pStat5 (pY694) (9351), pan-actin (4968). Sigma: b-actin AC-15 1/10000 (A5441). g-actin (cytoplasmic, lot1108 a gift from Christine Chaponnier and Michael Way). Abcam: mCherry (ab167453).
Antibodies used for in vitro stimulation: BD Biosciences: CD28(37.51) (553141), as indicated. CRUK Facility: CD3e (2C11) as indicated, Blocking/activating antibodies: eBioscience: IL-2 (S4B6) (16-7020-85), control Rat IgG2ak (eBR2a) (16-4321-85), IL-2 blocking JES6-(1A12) (503706), CD40 1C10 (16-0401-85).
Antibodies and dyes used for Immunofluorescence were purchased from: Biolegend: B220-AlexaFluor-488 1:100 (rat RA3-GB2). Invitrogen: Cell Tracker deep red 1uM (C34565), Cell Trace violet 5uM (C34557). Abcam: Pericentrin 1:500 (Ab4448).
Cytokines were purchased from: ImmunoTools: rmIL-2 (12340026), Chiron B.V rhIL-2 (Proleukin). R&D: rmIL-12 (419-ML-050).
Antibodies for extracellular markers were used at a dilution of 1:200 and purchased from: eBioscience: CD8 (53-6.7), CD122 (5H4), TCRb (H57-597), CD44 (IM7), CD5 (53-7.3), CD62L (MEL-14), CCR7 (4B12), NKG2D (CX5), LFA-1/CD11a (M17/4). BD biosciences: CD11b (M1/70), CD45.1 (A20), CD45.2 (104), CD4 (RM4-5), CD127 (SB/199), KLRG1 (2F1), TCRb (H57-597), CD132 (TUGm2), CD25 (PC61), CD69 (H1.2F3). Biolegend: CD11a/CD18 (H155-78), CD62L(MEL-14), CD69 (H1.2F3), CCL5 (2E9), B220 (RA3-GB2). Streptavidin Brilliant violet 421.Jackson Immunoresearch: APC goat anti-human IgG F(ab')2 fragments (109-135-098), R&D systems: rmICAM1-Fc (796-IC-050).
Antibodies for intracellular staining were used at a dilution of 1:200 unless stated otherwise, and purchased from: eBioscience: IRF4 (3E4), IFNg (XMG1.2), GranzymeB (NGZB), Fixable viability dye eFluor780 1:500 (65-0865-14). BD Biosciences: Bcl-2 (3F11), Caspase-3 (C92-605), pSTAT5(pY694) (47) (562077), panSTAT5 (89), (610192), TNF (MP6-XT22), BrdU FITC 1:50 (556028), CD16/32 FcBlock 1:100. Cell Signaling: pAKT(T308) (D25E6), pS6 240/244 (D68F8), pS6 235/236 (D57.2.2E), p44/42-ERK (D13.14.4E), pStat5 (pY694) (9351), Stat5 (D206Y) (94205).
Invitrogen: anti-DNaseI 488 1:200 (D12371 5mg/ml),Phalloidin Alexa Fluor 647 1:50 (A22287), CellTrace CFSE 4uM, SNARFTM 2.8uM.
Antibodies used for Western blotting were used at 1:1000 unless stated otherwise and purchased from: Santa-Cruz Biotechnology: anti-SRF (G20). BD Biosciences: panERK(16), panSTAT5 (89). Bethyl laboratories: MRTF-B (A302-786A). Biolegend: GAPDH 1/5000 W17079A (607901). Cell Signaling: pStat5 (pY694) (9351), pan-actin (4968). Sigma: b-actin AC-15 1/10000 (A5441). g-actin (cytoplasmic, lot1108 a gift from Christine Chaponnier and Michael Way). Abcam: mCherry (ab167453).
Antibodies used for in vitro stimulation: BD Biosciences: CD28(37.51) (553141), as indicated. CRUK Facility: CD3e (2C11) as indicated, Blocking/activating antibodies: eBioscience: IL-2 (S4B6) (16-7020-85), control Rat IgG2ak (eBR2a) (16-4321-85), IL-2 blocking JES6-(1A12) (503706), CD40 1C10 (16-0401-85).
Antibodies and dyes used for Immunofluorescence were purchased from: Biolegend: B220-AlexaFluor-488 1:100 (rat RA3-GB2). Invitrogen: Cell Tracker deep red 1uM (C34565), Cell Trace violet 5uM (C34557). Abcam: Pericentrin 1:500 (Ab4448).
Cytokines were purchased from: ImmunoTools: rmIL-2 (12340026), Chiron B.V rhIL-2 (Proleukin). R&D: rmIL-12 (419-ML-050).

Validation

Primary antibodies were titrated to determine optimal concentration. For validation of antibodies and to establish specificity of staining for activation markers, expression levels of the antigen of interest were compared in the same cells without (negative control) and with (positive control) activation. In some experiments, specificity of the primary antibody could be established directly in the same staining sample, such as in adoptively transferred mice with CD8 T cells subsets. In these experiments level of expression

of the activation marker was compared between adoptively transferred CD8 T cells (positive control) and bystander endogenous CD8 T cells acting as negative control. In other experiments, cells stained with a specific primary antibody was compared to cells stained with it's respective isotype control antibody as negative control.

Animals and other research organisms

Policy information about studies involving animals; ARRIVE guidelines recommended for reporting animal research, and Sex and Gender in Research

Laboratory animals	C57BL6/J mice were Elk4-/-(SAP-1 null), Costello, P.S. et al., Nat Immunol 5, 289-298 (2004).; R26CreERT2 (tm9(cre/ESR1)Arte, Seibler, J. et al., Nucleic Acids Res 31, e12 (2003).; conditional Srff/f, Parlakian, A. et al.,Mol Cell Biol 24, 5281-5289 (2004).; conditional Mrtfa-/-Mrtfbf/f, Mokalled, M.H. et al., Development 137, 2365-2374 (2010) and transgenic Lifeact-EGFP, Riedl, J. et al., Nat Methods 7, 168-169 (2010). Srff/f Tam-Cre (CD45.2), WT Tam-Cre (CD45.1), Mrtfa-/-Mrtfbf/f TamCre (CD45.2), Elk4-/- and Lifeact-EGFP animals were crossed to OT-I TCR transgenics (Tg(TcraTcrb)1100Mjb/Crl). OT-I Lifeact-EGFP animals were further crossed to Mrtfa-/-Mrtfbf/f TamCre. Rag2-/- (RAG2 tm1Fwa). All genetically modified mouse lines were back crossed to C57BL/6J. Young adult female mice were used aged between 8-12 weeks old. The housing conditions are the following: light cycles fluctuate from 7am to 7pm.Temperature range from 20-24 degrees C and humidity is 55%+/- 10%.
Wild animals	No wild animals were used in this study.
Reporting on sex	xperiments presented in the paper were performed with female mice generally between 8-12 weeks unless specified in the text. The same observations were confirmed in male mice for preliminary experiments.
Field-collected samples	No field collected samples were used in this study.
Ethics oversight	All experiments were performed in accordance with the United Kingdom Animals(Scientific Procedures) Act of 1986. The UK Home Office accredited all researchers for animal handling and experimentation. Dispensation to carry out animal research at the Francis Crick Institute was approved by the Institutional Ethical Review Body and granted by the UK government Home Office. Animals were maintained under specific-pathogen–free conditions in The Francis Crick Institute UK Biological Resources Facility. Animal experimentation was carried out under Home Office licences PPL PP0389970, P7C307997, 80/2602 and 70/7982.

Note that full information on the approval of the study protocol must also be provided in the manuscript.

Plants

Seed stocks	*Report on the source of all seed stocks or other plant material used. If applicable, state the seed stock centre and catalogue number. If plant specimens were collected from the field, describe the collection location, date and sampling procedures.*
Novel plant genotypes	*Describe the methods by which all novel plant genotypes were produced. This includes those generated by transgenic approaches, gene editing, chemical/radiation-based mutagenesis and hybridization. For transgenic lines, describe the transformation method, the number of independent lines analyzed and the generation upon which experiments were performed. For gene-edited lines, describe the editor used, the endogenous sequence targeted for editing, the targeting guide RNA sequence (if applicable) and how the editor was applied.*
Authentication	*Describe any authentication procedures for each seed stock used or novel genotype generated. Describe any experiments used to assess the effect of a mutation and, where applicable, how potential secondary effects (e.g. second site T-DNA insertions, mosiacism, off-target gene editing) were examined.*

Flow Cytometry

Plots

Confirm that:

☒ The axis labels state the marker and fluorochrome used (e.g. CD4-FITC).

☒ The axis scales are clearly visible. Include numbers along axes only for bottom left plot of group (a 'group' is an analysis of identical markers).

☒ All plots are contour plots with outliers or pseudocolor plots.

☒ A numerical value for number of cells or percentage (with statistics) is provided.

Methodology

Sample preparation	Tissues including lymph node and spleen were disaggregated through a 70um nylon mesh in cold RPMI-1640 10% FCS. Blood was collected into Sarstedt EDTA KE/1.3 tubes before red blood cell lysis. Cells were stained in ice-cold FACS buffer (1% FCS, 2mM EDTA in PBS) with combinations of fluorochrome-conjugated antibodies.<br>For intracellular staining, cells were fixed and permeabilised using eBioscience intracellular fixation and permeabilisation kit (BD Biosciences). For pSTAT5 and pERK staining, cells were fixed in 2% paraformaldehyde, washed and permeabilised in ice-cold methanol for 30 min, washed twice in PBS, 10% FCS and stained for 1 h.

Instrument	BD-Fortessa Instruments (BD Bioscience) for analysis, BD INFLUX or FACS ARIA-III for sorting cells
Software	FlowJo 9.9 software (BD Bioscience), BD FACS Diva software
Cell population abundance	Where indicated, T-cells were sorted to >97% purity as judged by cell surface marker expression
Gating strategy	Cells were gated as followed: 1. live cells, 2.FSC/SSC profile for lymphocyte, 3. doublet discrimination by FSC/SSC-W or FSC/FSC-H, 4. CD8, 5.CD45.1 and CD45.2, 6.marker of interest. Generally, boundaries for positive and negative populations are defined by comparing cells expressing the antigen to cells that do not express the antigen of interest. In cases where populations are represented as a bimodal distribution, we show percentages of cells expressing the antigen of interest. Other antibodies reveal a normal distribution for which the Median Fluorescence Intensity (MFI) is being showed.

☐ Tick this box to confirm that a figure exemplifying the gating strategy is provided in the Supplementary Information.
