# Supplementary material for: IL-2 delivery to CD8+ T cells during infection requires MRTF/SRF-dependent gene expression and cytoskeletal dynamics
Source: Nat Commun. 2024 Sep 11;15:7956. doi: 10.1038/s41467-024-52230-8 (PMC11391060; doi:10.1038/s41467-024-52230-8)

Source data: Fig 1B, 2B, 3E

Fig1B

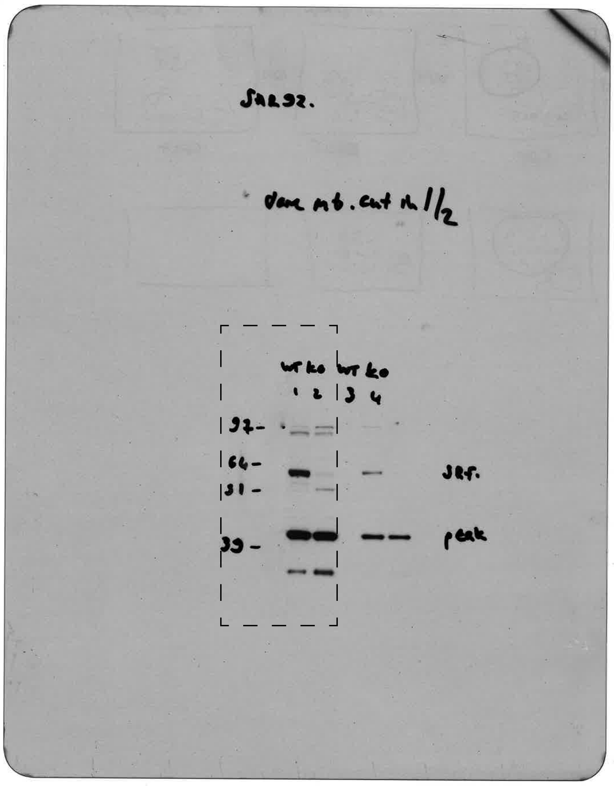

Fig1B and 2B and 3B:
the samples were run on the same gel
and transferred together on the same membrane
which was then cut in half for antibody staining

Fig 3E

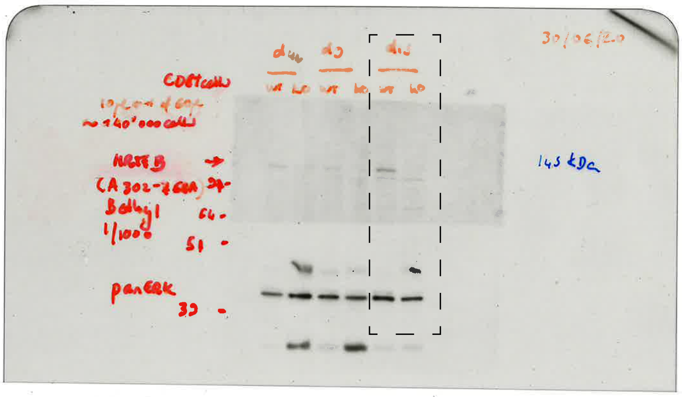

Fig 2B

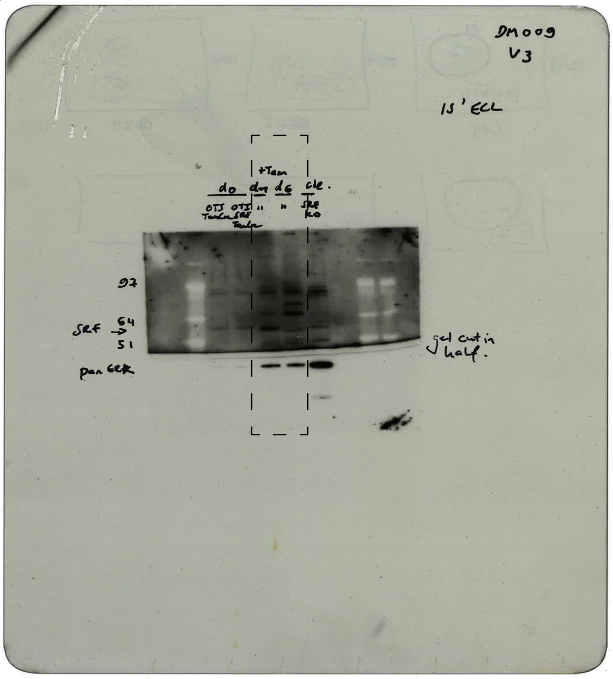

Source data: Fig 7B, 8F

Fig 7B

pstat5 is probed on the same membrane as panERK and
Stat5 is probed on the same membrane as panERK

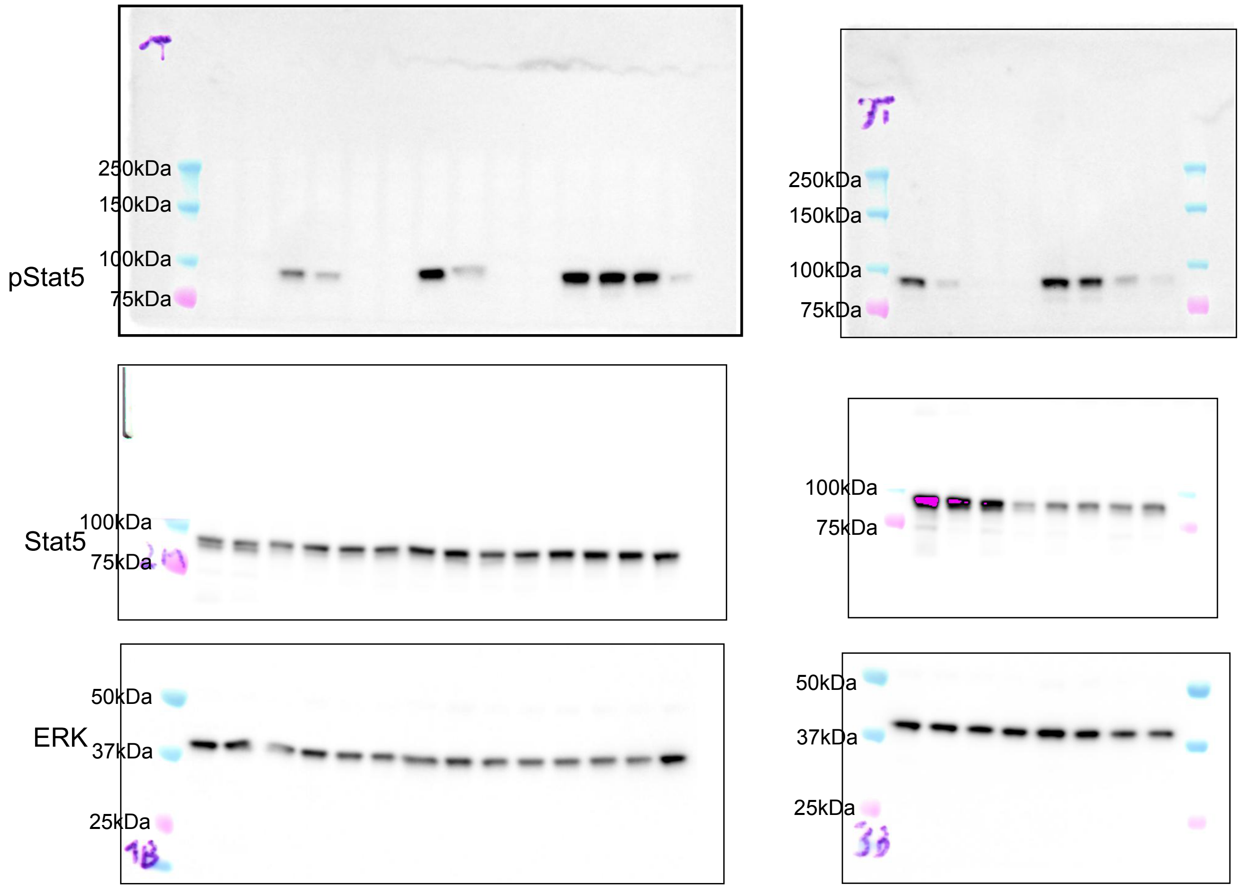

Fig 8F

β-actin and pan actin and Gapdh are probed on the same membrane
and γ-actin and gapdh are probed on the same membrane

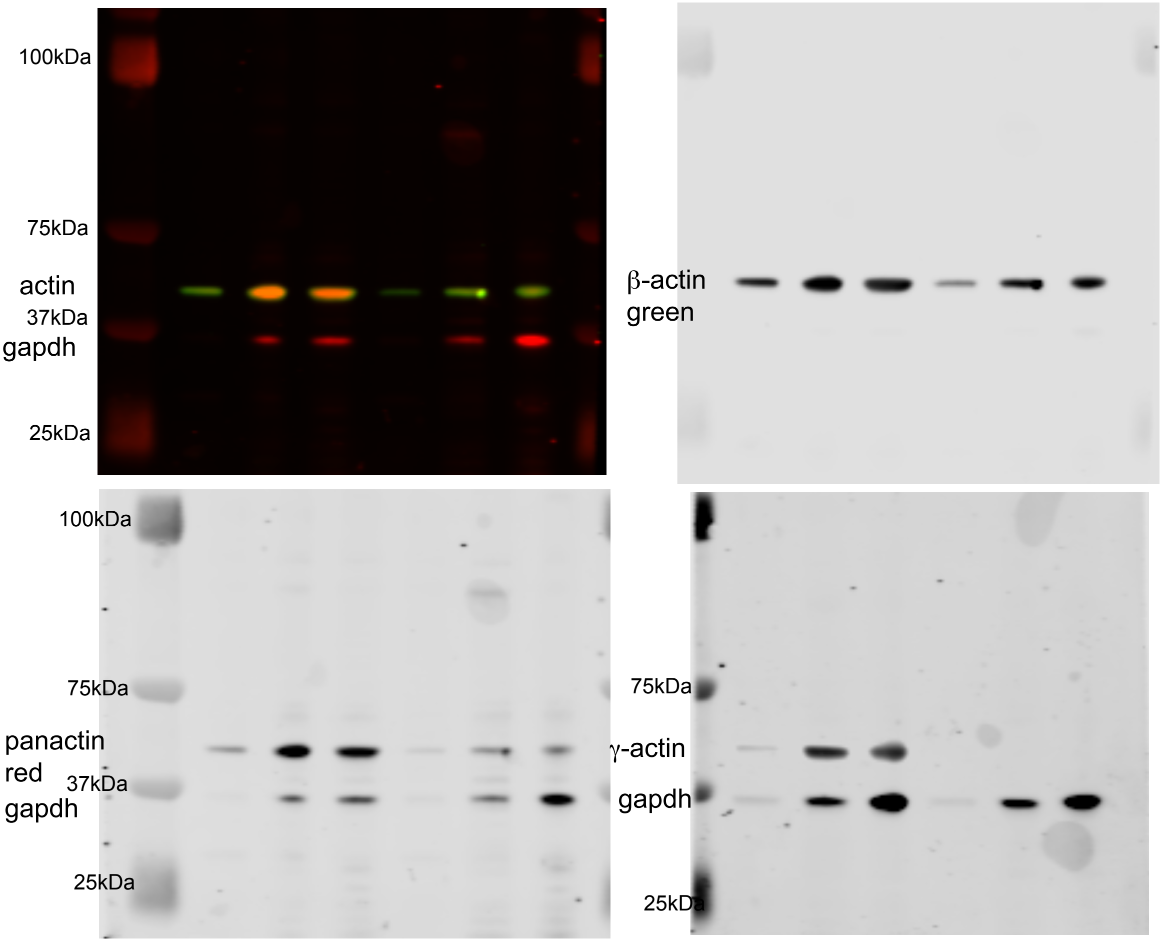

Source data: Fig 9B, 9G

Fig 9B

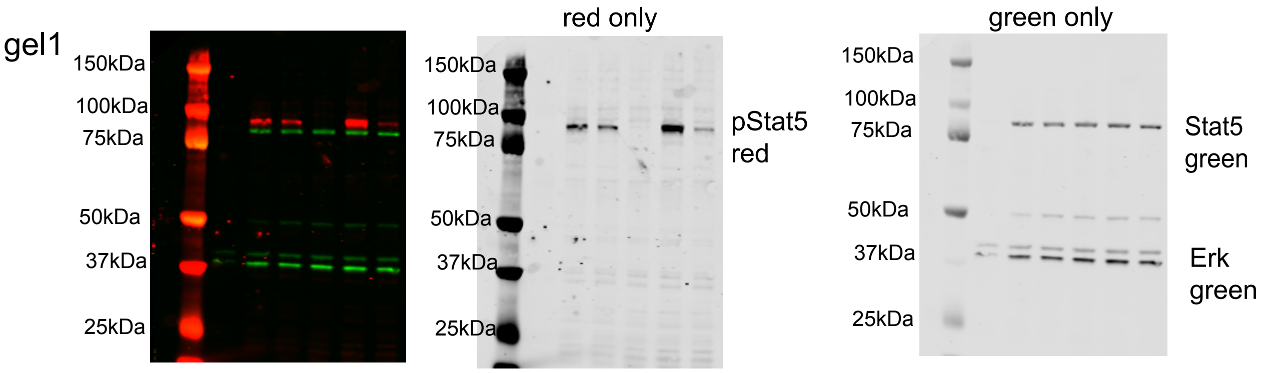

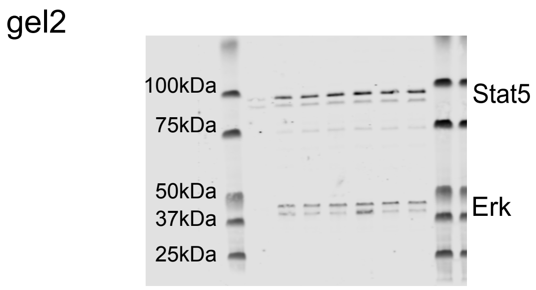

Antibodies for pStat5,Stat5 and Erk are probed on the same membrane (gel1)
Stat5 Ab is run together with pan ERKAb (gel2)

Fig 9G

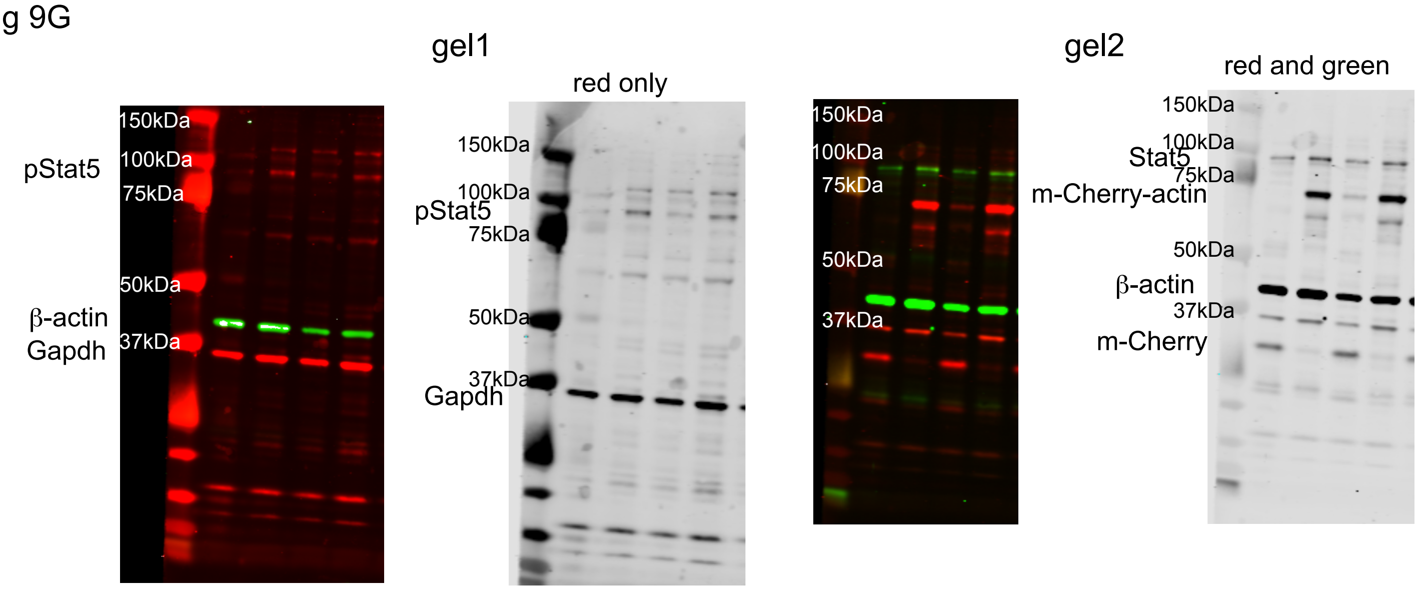

Antibodies for pstat5, β-actin and Gapdh are probed on the same membrane (gel1)
Antiboides for stat5 ,m-cherry and β-actin are probed on the same membrane (gel2)

Source data: Supplementary Figure S9

Fig. S9A

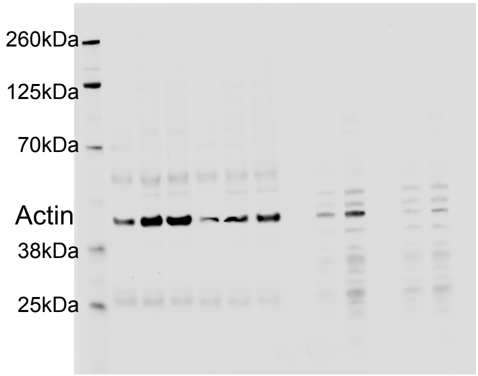

Fig. S9B

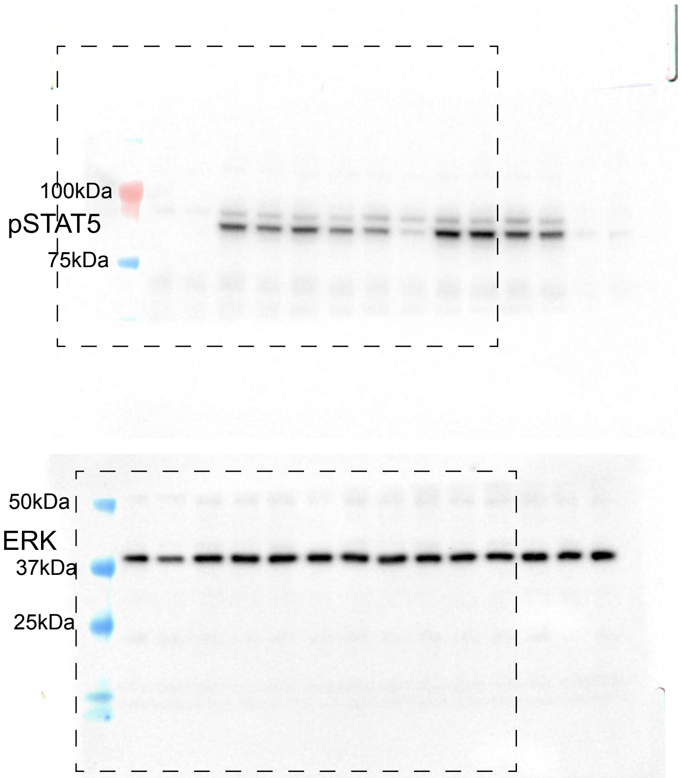

Supplement: Supplementary file 7 — Source Data [file 41467_2024_52230_MOESM7_ESM.zip › Zip source data files/Source Data File 2.pdf]
